# Testing Communication Concepts on COVID-19 Contact Tracing Among Black and Latinx/Hispanic People in the United States

**DOI:** 10.1007/s40615-021-01167-5

**Published:** 2022-04-07

**Authors:** Sandra Mullin, Shuo Wang, Irina Morozova, Julia Berenson, Nana Asase, Denene Jonielle Rodney, Sharon Arthur, Nandita Murukutla

**Affiliations:** 1 Vital Strategies, 100 Broadway, 4th Floor, New York, NY 10005 USA; 2 Zebra Strategies, 421 Seventh Avenue, Suite 1100, New York, NY 10001 USA

**Keywords:** COVID-19, Contact tracing, Mass media campaign, Racial and ethnic disparities, Black, Latinx/Hispanic

## Abstract

**Objective:**

Black and Latinx/Hispanic people were more than twice as likely to die from COVID-19 than White people, but because of legacies of discrimination and maltreatment in health care, were less likely to participate in some public health responses to COVID-19, including contact tracing. This study aimed to test three communication campaign concepts to engage Black and Latinx/Hispanic people in contact tracing efforts.

**Methods:**

Twelve focus group discussions with 5 to 10 participants each were conducted online among participants from Black and Latinx/Hispanic urban populations in Philadelphia and New York state. Participants provided sociodemographic information and were presented with potential campaign concepts and prompted to rate the concepts and engage in open-ended discussion. For rating and sociodemographic data, chi-square tests were performed. For open-ended discussion data, a thematic analysis approach was used.

**Results:**

Across groups, the campaign concept that was rated most likely to encourage cooperation with contact tracing efforts was “Be the One,” with 45% of total first-place votes. Participants expressed that the campaign caught their attention (79%), motivated them to engage with contact tracers (71%) and to talk to others about contact tracing (77%). Discussions also elucidated: the importance of community engagement; the need for clearer explanations of contact tracing; the preference for already trusted, community-based contact tracers; the need to reassure people about confidentiality; and for contact tracing to be culturally competent and empathetic.

**Conclusions:**

This study highlights how strategic, culturally sensitive communication can buttress current and future contact tracing efforts, especially among Black and Latinx/Hispanic people.

## Introduction

In the United States (U.S.), people in communities of color are at disproportionate risk of infection, hospitalization, and death from COVID-19 [1–8]. Non-Hispanic Black and Latinx/Hispanic people are more than twice as likely than White people to die from COVID-19 in many parts of the United States [8–13]. Similarly, adults with low incomes have disproportionately higher rates of chronic conditions that increase risk of serious illness if infected with COVID-19 [14, 15]. These disparities in the COVID-19 pandemic are recent manifestations of long-standing systemic health and social inequities in the United States [16]. Given the pandemic’s unequal impact on people in communities of color and with low incomes, public health programs and policies must pay special attention to the needs of these people [17].

Many people in communities of color, and Black people in particular, have a deep mistrust of the health system and government that is a result of multiple factors: current and historical interpersonal and structural racism; the legacy of Tuskegee and other exploitative medical research; low representation of people of color in medical professions; and health disparities that have persisted for centuries [18–29]. Recent studies have found that Black and Latinx/Hispanic people are more likely to mistrust medical and public health authorities, have false information, be reluctant to participate in contact tracing, and be skeptical of a vaccine for COVID-19, compared with White people [25–39]. To address COVID-19 health inequities, it is important for public health and medical professionals to communicate information about contact tracing, vaccines, and other public health interventions in a manner that regains trust of people in communities of color [40].

### Contact Tracing Efforts in the the United States

Contact tracing—the process by which public health agencies identify people with an infectious disease and those they came into contact with—is an established public health tool to prevent the spread of infectious diseases, especially when vaccinations and treatments are limited [41–43]. According to the Centers for Disease Control and Prevention, contact tracing is most effective when part of a multifaceted response to an outbreak that includes other infection prevention and control measures, such as testing and vaccination [44]. It is a particularly useful tool to stem the spread of COVID-19 in areas with low vaccination rates and can be used to create linkages to vaccination and other services [45, 46]. While early in the pandemic contact tracing was recognized as a key intervention to reduce the spread of COVID-19 [47–53], with the introduction of vaccines, many states began to shift focus and resources away from contact tracing efforts [54]. However, particularly given uneven vaccination rates and the introduction of newer, more contagious variants of COVID-19, contact tracing remains an important tool to contain the spread of the virus [54]. Contact tracing is also routinely used, along with vaccines and treatments, to contain diseases including tuberculosis, measles, and various sexually transmitted diseases [54]. Therefore, understanding how to engage people in contact tracing is an important ongoing issue for the COVID-19 pandemic, as well as the other infectious diseases in our midst and those to come.

Contact tracing only works if people participate and provide information about who they have been in contact with, so it requires confidence that authorities will guard personal information and protect privacy. Therefore, contact tracing efforts should engage trusted community stakeholders such as faith leaders, and use communication that takes into account local context and culture in order to generate public support and confidence [55, 56].

Public health media campaigns, appearing on television, radio, social media, in print, and on out-of-home channels such as billboards or posters, can also play a significant role in contact tracing [57, 58]. When campaigns are done well, including pretesting with audiences, they can raise perception of personal risk, shift attitudes, and eventually help to change behaviors [59–61]. Mass media campaigns that celebrate and embrace cultural contexts of the community can empower and encourage community members: consideration of cultural aspects such as language, ethnic background, religion and cultural practices, reinforce powerful community connections and improve message dissemination [62, 63]. In addition, mass media campaigns in combination with strategies to engage communities, such as by involving community leadership, civic and voluntary groups, and organizations, have been found to be effective in health behavior change [64].

In order to design public health media campaigns that resonate with people in communities of color in the U.S. and increase people’s participation in contact tracing efforts, Vital Strategies, a global health organization, in partnership with The Soze Agency, a strategic communications firm, and Zebra Strategies, an ethnic market research company, developed and tested multiple advertising concepts. Three concepts with varied creative execution styles were developed with the objective to encourage people to participate in contact tracing. The concepts were tested in focus groups made up of members of the priority audience to assess the potential effectiveness of the concepts in building community engagement and trust and increasing intentions to participate in contact tracing efforts. The study also tested the same concepts among leaders in these communities. This paper summarizes the key findings from this study, including beliefs about contact tracing and features of the messaging that can strengthen trust and encourage willingness to engage in public health efforts.

## Methods

### Test Material

Table 1 describes and presents examples of stills from the three concepts tested: (1) “Spread the Love,” (2) “Keep in Contact,” and (3) “Be the One.” The three concepts used different stimuli tailored to local features (e.g., for Philadelphia, the image included a person wearing a Philadelphia Eagles shirt). Each concept was shown to participants in mock-ups of print, social media, and radio advertisements.

**Table 1:**
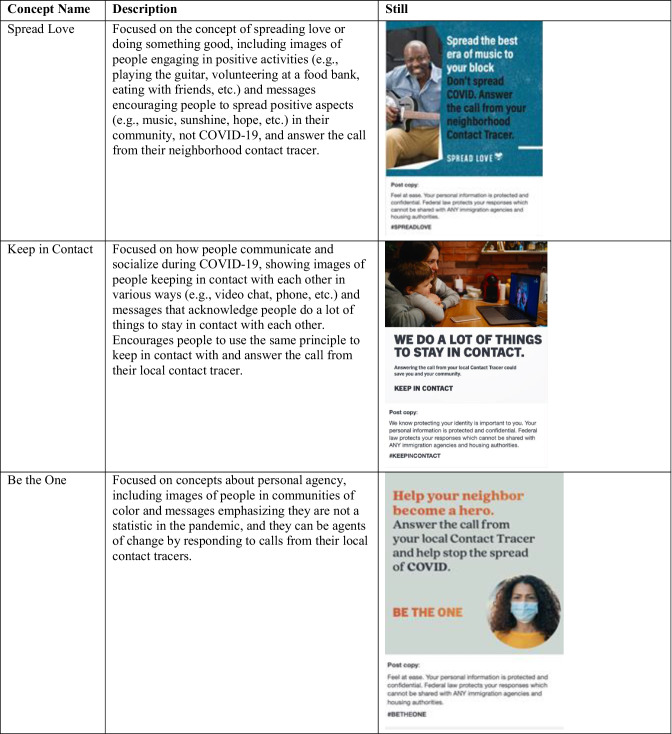
Campaign concepts

### Participants and Setting

Primary participants included people who: (1) reside in New York state or Philadelphia; (2) self-identify as Latinx/Hispanic, African American, Black^1^ or Asian; (3) have household income lower than US $60,000; and (4) are age 18 and above. Secondary participants included people who: (1) are key opinion leaders and influencers in the primary participants’ communities; (2) are age 18 and above; (3) and self-identify as African American, Latinx/Hispanic or White Caucasian. A convenience sample was used to recruit participants. A participant screener was developed to capture the demographic information of community members living in high-risk areas in New York and Philadelphia who were part of Zebra Strategies’ network. Secondary participants were social service, community educators and outreach advocates who were recruited by the researchers because of their work within agencies and organizations in direct proximity to the communities represented by the primary participants. A separate participant screener was used to identify these community stakeholders.

We used focus group methodology to encourage the discussion of issues about COVID-19 and contact tracing, and ratings and reactions to potential campaign concepts [65]. Twelve online focus groups consisting of 5 to 10 participants each were held. Eleven groups were held for primary participants (community members and likely service recipients) and one group was held separately for secondary participants (community influencers). Focus groups were kept small in an effort to encourage participants to be more comfortable discussing sensitive issues [66]. Recruitment was conducted by telephone. All focus group participants were provided written informed consent before beginning the study and were given a modest monetary incentive for their participation.

Participants were assigned to groups based on date, location, language, and self-identified ethnic background. Two focus groups were conducted in Spanish and 10 were conducted in English. Each focus group was led by trained facilitators and lasted approximately 90 min. Focus groups were held online on the Civicom conferencing platform, with audio and video recordings, between June 22 and June 26, 2020. The focus groups were conducted online to avoid the risks of in-person gatherings during the COVID-19 pandemic.

### Discussion Guide and Data Collection Procedures

An evidence-informed discussion guide was developed to provide structure for each focus group discussion, enabling consistency and comparability across groups. Topics, questions and prompts covered in the discussion guide are listed in Table 2. Each focus group began with general discussion about participants’ experiences during the pandemic, then continued with both quantitative ratings of the concepts and discussions about the concepts. The same process and materials were used for both primary and secondary participants.

**Table 2:** Discussion guide topics, questions, and types

Topics	Questions	Types
*Introduction and understanding of COVID-19*		
Introduction	Let’s go around and introduce ourselves. Please tell me… o Your first name o City/Neighborhood you live in	Open-ended
Overall status and concerns during the COVID-19 pandemic	We understand that this is a strange time for many of you and again we thank you for joining us. How is everyone doing now? What are your biggest concerns currently? Safety? Food insecurity? Housing? Job? Health care? Illness? What else?	Open-ended
Knowledge of COVID-19	What is your understanding of COVID-19?	Open-ended
Infection and testing	Were you, your family, or anyone you know infected? Were you tested? Why did you decide to get tested? Why not?	Open-ended
Trusted sources for information about COVID-19	Let’s talk about ‘trust,' who do you trust to give you information and advice about the pandemic? (Probe: clergy, government, family member, health department, celebrities etc..)	Open-ended
Understanding of contact tracing	Have you heard about contact tracing? What is your understanding of contact tracing? Good, bad, why we need it? How it’s done? Probe: what they know	Open-ended
*Concept testing*		
Individual ratings for each concept	This campaign caught my attention. Yes or No? I will talk with others about contact tracing after seeing this campaign. Yes or No? This campaign made me motivated to talk with contact tracers. Yes or No?	Poll
Comparative ratings across all concepts	Which one of the campaigns is most eye-catching? Which one of the campaigns would most likely make you talk with others about contact tracing? Which one of the campaigns would most likely make you motivated to talk with contact tracers? Which one of the campaigns gives the best understanding of the benefits of contact tracing participation?	Poll
Reactions for each concept	What did you think about this concept/idea? What was your first impression/reaction? What do you like about it? Dislike about it? Any new information? What is the main message of the concept? What is it trying to communicate to you? Main ask? Was it relevant to you and your community? Does this apply to [New Yorkers? Philadelphians?] Does it apply to someone you might know? Would you trust the information? Why or why not? If you saw this on TV, online, on the subway or bus shelter, or in newspapers, would it catch your attention? What does/doesn’t work? Probe: What did you think of the images? What do you think of the individuals pictured? Words? Colors? Design? Tone-how you would describe the tone? Is there anything confusing about this concept/idea or the language used? If so, what? How would you more clearly explain the concept/idea in your language? Are there specific words or phrases that should be clarified? Does this concept make you want to learn more about this? What do you think is missing? Is there anything you would suggest changing? Could you imagine yourself or someone you know participating in a contact tracing program? Do you feel you might be more open if the campaign asked you to help contact tracers?	Open-ended
Best overall concept and why	Now, take a moment and write down: Which of these concepts gave you the best understanding of the benefits of participating in contact tracing? What did you learn from these concepts? When you think about this information, which of these concepts/ideas made it easier to understand? What does this concept do well?	Open-ended
*Contact tracing*		
Willingness to participate in contact tracing	If you tested positive for COVID-19 would you participate in a contact tracing program? If you suddenly got a call from a health department, what would be your reaction? Why? Have you or anyone you know participated in a contact tracing program? What was the experience? Were there any barriers? (Probe: language, money, work schedule, childcare)	Open-ended
Trusted sources for information about COVID-19	Earlier, we talked about “trust”, who do you trust to give you information and advice about the pandemic? (Probe: clergy, government, family member, health department, famous people etc.)	Open-ended
Knowledge and perceptions of contact tracers and trusted sources for contact tracing	What do you know and think of people behind contact tracing (often called contact tracers)? Who would you trust to conduct a contact tracing program in your community?	Open-ended
Information seen about contact tracing and where it was seen	What information have you seen about contact tracing and where did you see it (television, radio, social media, posters/flyers, etc.)?	Open-ended
Top trusted sources for information about COVID-19 and contact tracing	What are your top 3 trusted sources (trusted people) for receiving information on COVID-19? o Community organization? Which one(s): ___________________ o Government website? Which one(s):___________________________ o Word of mouth (i.e. Texts, Emails, Online news source) o Not interested in more information o Other Of these, which one would you trust to get more information about contact tracing?	Poll/Open-ended
*Wrap-up*		
Additional questions	Any additional questions to ask?	Open-ended

The guide included several questions and prompts designed to elicit conversation about experiences with COVID-19 and contact tracing, such as knowledge about the pandemic and trusted sources for information about it. The guide then instructed the facilitator to show participants the three concepts. After showing each concept, the facilitator conducted a poll about that concept using the online platform’s polling function, and asked open-ended questions to assess participants’ ratings and reactions. The order of presentation of the three concepts was randomized across groups to minimize order effects.

Prior to the discussion, data were also collected on participants’ demographic characteristics, including gender, age, ethnic background, income, education, employment, marital status and parental status.

The order of presentation of the findings below does not necessarily follow the order in the discussion guide but is set up to aid readability.

### Ethics

The study was evaluated and approved under applicable U.S. regulations related to human subjects research by the Vital Strategies Human Protections Administrator.

### Data Analysis

Systematic analytic procedures were applied for both quantitative and qualitative data. For quantitative polling and sociodemographic data, frequencies and chi-square tests were performed. For qualitative data, focus group discussions were transcribed verbatim. Spanish transcripts were translated and transcribed by professional services. To check for transcription accuracy, a review of the transcripts and audio recordings was conducted. All transcripts were systematically coded manually, identifying themes and sub-themes across all focus groups in an iterative process over multiple readings of the material [67]. IBM SPSS v25 statistical software was used to code and analyze the data.

For qualitative data, we conducted open coding to identify beliefs about contact tracing and identify common themes related to challenges/opportunities. We assessed qualitative discussions about the tested concepts, identifying five distinct themes for reactions to concepts: (1) attention; (2) comprehension; (3) motivation; (4) personal relevance; and (5) cultural appropriateness. Table 3 describes each theme, its definition, and example quotes from participants. We examined cross-group differences in themes; major differences were not observed. Themes were also similar across key populations of interest, but with some noted differences for Latinx/Hispanic participants. As there were no significant differences between groups, data for the primary and secondary participants were combined and reported in the aggregate; when relevant, observations for Latinx/Hispanic participants were further elaborated.

**Table 3:** Themes, descriptions, and examples from open-ended discussions about concepts

Themes	Description	Example quote from open-ended discussions about concepts*
Attention	Does the concept attract audience attention? Is the concept memorable?	“This did not get my attention as what this is for is not in the first few words. I think the smaller lettering of the point of the ad made me not want to read the text.” (Keep in Contact) “It was not very memorable advertisement; it was actually boring and some of the ads were not relevant.” (Keep in Contact) “They were aesthetically pleasing and very positive.” (Spread Love) “I feel like instead of spreading COVID, the virus, you’re actually spreading love. I just really like the wordplay with that. That caught my eye.” (Spread Love) “This more than any of the other ones, there was a sense of urgency.” (Be the One) “The wording was specific and direct.” (Be the One)
Comprehension	Is the concept is clearly understood?	“What is the message here? If contact tracing is the theme it missed the mark. These ads don't explain what it is.” (Keep in Contact) “I know there’s a subliminal message towards it because it’s trying to talk about social distancing basically, but I feel like it doesn’t really talk about the impact of stopping the spread of COVID-19 very explicitly.” (Keep in Contact) “I would have to obtain more information.” (Spread Love) “I just don’t get, hey, look at this ad if you want to have some vital information about COVID and how you can look out for your loved ones and contact tracing.” (Spread Love) “The campaign was trying to make it easy to understand.” (Be the One) “It is informative and straight to the point.” (Be the One)
Motivation	Does the concept inspire participants to take a desired action?	“Not really [motivated to talk with others about contact tracing]. I would probably reach out to family but it may not be about contact tracing.” (Keep in Contact) “I would talk to contact tracers to help the community and make people aware.” (Keep in Contact) “This ad doesn't make me want to do more than I have before.” (Spread Love) “No…..It didn’t motivate me very much.” (Spread Love) “This more than any of the other ones, there was a sense of urgency…you’ve got to answer this call to save lives in your community.” (Be the One) “It invites me to take action, which inspires me to ask others to join the cause.” (Be the One)
Personal Relevance	Can participants connect with the concept? Does it take their point of view into consideration?	“This campaign was very relatable, it connects with the viewer, people can relate to each ad.” (Keep in Contact) “I feel like ‘Keep in Contact’ was showing you pictures of what our life is like now. We’re on the phone having Zoom conferences as opposed to face-to-face. You can’t go see your grandparents, or doing a drive-by birthday party. This is just what life is now and…what it looks like for the foreseeable future.” (Keep in Contact) “I liked the local feeling of the ad.” (Spread Love) “It seems to focus on the brotherly love / sisterly affection theme for Philadelphia.” (Spread Love) “A lot of people can feel helpless during this time. By "being the one" it can help people feel like they are doing something.”(Be the One) “It is letting me know that I can help by just being that one person to help someone else be aware and that person will help the next.” (Be the One)
Cultural Appropriateness	Is the concept consistent with cultural values, attitudes, beliefs, traditions, and history of participants?	“It pulls from family ties and how at the time is more important to stay in contact.” (Keep in Contact) “The contact piece really hit home and was relevant to picking up the call and what many have been doing day to day to stay in contact with friends and family.” (Keep in Contact) “The ads were culturally diverse.” (Spread Love) “It seems to target specifically minorities in each ad. That could possibly offend.” (Spread Love) “It’s about caring about your community…how we can all come together and help each other.” (Be the One) “[It] seemed to target a specific demographic as far as race and economic status as if they're the only ones infecting others.” (Be the One)

^*^Note: for each quote, the concept discussed is listed in parentheses

Participants’ ratings of the concepts were captured via polling on the online platform. Individual polls asked participants to rate each concept on three parameters of appeal: (1) caught their attention; (2) encouraged them to talk with others about contact tracing; and (3) motivated them to talk with contact tracers. Comparative polls asked participants to select the best of the three concepts on four parameters of appeal: (1) caught their attention; (2) encouraged them to talk with others about contact tracing; (3) motivated them to talk with contact tracers; and (4) gave the best understanding of the benefits of participating in contact tracing.

The top concept was found by triangulating among the multiple measures used in the study, including reactions to the concepts on the individual quantitative measures, the comparative polls, and the qualitative discussions. The concept that rated most highly on all three sets of measures was determined to be the most effective.

## Results

### Demographic Characteristics

Table 4 presents demographic characteristics of the study sample (n = 89). Among all participants across the 12 focus groups, nearly 60% identified as female, 38% as male, and 2% as other. Participants across all focus groups were aged 25 to 77 years. When asked to self-identify their ethnic background, 63% of all participants identified themselves as non-Hispanic Black or African American, 24% as Latinx/Hispanic, 2% as Asian American/Asian, and 8% as other. The majority of participants earned below $40,000 per year: 72% of participants reported their annual income was $30,000 to $40,000 and 17% reported their annual income was less than $30,000. Of the rest, 3% reported an annual income between $50,000 and $60,000, and 7% reported an annual income of more than $60,000.

**Table 4:** Participant demographics

	All participants		Primary participants		Community stakeholders	
	Count (N)	Percent (%)	Count (N)	Percent (%)	Count (N)	Percent (%)
Gender						
Female	53	59.6	48	60.8	5	50.0
Male	34	38.2	30	38.0	4	40.0
Other	2	2.2	1	1.3	1	10.0
Age						
18–35	28	31.5	27	34.2	1	10.0
36–55	46	51.7	39	49.4	7	70.0
55–77	15	16.9	13	16.5	2	20.0
Ethnic background						
Non-Hispanic White/Caucasian	3	3.4	0	0.0	3	30.0
Non-Hispanic Black/African American	56	62.9	50	63.3	6	60.0
Latinx/Hispanic	21	23.6	20	25.3	1	10.0
Asian American/Asian	2	2.2	2	2.5	0	0.0
Other	7	7.9	7	8.9	0	0.0
Annual income						
Less than $20,000	1	1.1	1	0.0	0	0.0
$20,000–$30,000	14	15.7	14	0.2	0	0.0
$30,000–$40,000	64	71.9	64	0.8	0	0.0
$40,000–$50,000	0	0.0	0	0.0	0	0.0
$50,000–$60,000	3	3.4	0	0.0	3	30.0
More than $60,000	6	6.7	0	0.0	6	60.0
Missing	1	1.1	0	0.0	1	10.0
Education						
Less than high school	2	2.2	2	0.0	0	0.0
High school	26	29.2	26	0.3	0	0.0
Vocational or technical school	1	1.1	1	0.0	0	0.0
Attending or some college	19	21.3	19	0.2	0	0.0
College graduate	27	30.3	24	0.3	3	0.3
Postgraduate	14	15.7	7	0.1	7	0.7
Employment						
Full-time	52	58.4	42	0.5	10	1.0
Part-time	15	16.9	15	0.2	0	0.0
Self-employed/Own a business	1	1.1	1	0.0	0	0.0
Homemaker	2	2.2	2	0.0	0	0.0
Student	2	2.2	2	0.0	0	0.0
Unemployed	11	12.4	11	0.1	0	0.0
Retired	4	4.5	4	0.1	0	0.0
Unable to work	1	1.1	1	0.0	0	0.0
Missing	1	1.1	1	0.0	0	0.0
Marital status						
Married	33	37.1	28	35.4	5	50.0
Widowed	2	2.2	2	2.5	0	0.0
Divorced or separated	7	7.9	6	7.6	1	10.0
Never married	4	4.5	4	5.1	0	0.0
Single	40	44.9	36	45.6	4	40.0
Living with significant other	3	3.4	3	3.8	0	0.0
Any children						
No	30	33.7	27	34.2	3	30.0
Yes	59	66.3	52	65.8	7	70.0

### Beliefs About Contact Tracing: Challenges and Opportunities

Contact tracing was understood by participants in the abstract but few had firsthand experience or deeper understanding about what it is, how it works, and who it might help. At the time of the focus groups in June 2020, no one recalled knowing anyone who had participated in COVID-19 contact tracing, and few recalled seeing or hearing communication on mass media about it. For older respondents, memories of contact tracing during the height of the HIV epidemic in the U.S. shaped their current understanding of the endeavor. Younger respondents perceived it as being linked to technology, such as a GPS-like system that digitally tracks people on their phones and sends alerts when they are close to someone with COVID-19. One participant said:

“It has to do with, like, GPS tracking, like with your phone and your computer, or something like that, something that’s a little bit more technological to it to see literally where you’ve been and who you were in contact with.” (Philadelphia; African American, Latinx/Hispanic, and Other Ethnicity).^2^

One of the most common themes from the open discussion about contact tracing was a general sense of mistrust, fear and suspicion:

“It causes fear…it’s the same thing as if somebody called you in the police precinct saying, ‘Come here, we want to talk to you.’ It’s like the same thing…” (New York City; Latinx/Hispanic).

Concern about possible surveillance, coercion, and privacy infringement permeated the conversation about contact tracing, with many participants wondering where the information collected by a contact tracer would go, with whom it would be shared, and how it would be used:

“I understand they’re trying to get an understanding of the spread and find out people who have it, but I just don’t understand who’s using the information, who’s not using it, and what are they using it for besides obviously COVID.” (New York City; African American).

In particular, participants expressed skepticism with contact tracers because of their link with the government:

“I’m very distrustful at this time of the government and what they’re going to do with information and data.” (Philadelphia; African American, Latinx/Hispanic)

There were also fears that the information gathered by contact tracers could be used to report information to immigration agencies, deny insurance or unemployment benefits or to take away civil liberties:

“I don’t know anybody who would trust that information won’t be shared with immigration agencies because I don’t know that anybody trusts the government much at this point.” (New York City and Philadelphia; African American, Latinx/Hispanic, and White Caucasian).

Similarly, participants expressed concern about talking to strangers (especially when sensitive medical information is being shared), and said they might not answer a call if they don’t recognize the caller ID:

“I don’t answer phone calls of people I don’t know. I’d just think it’s a telemarketer or a bill collector.” (New York City, African American)

Furthermore, there was a shared concern that contact tracing is about “snitching,” and that participation could stigmatize those who have COVID-19 and possibly expose them to social ostracization.

Yet, despite these concerns, participants said they would, under the right conditions, participate in contact tracing because they would want to play their part to help control COVID-19 for their families and community:

“I would talk to contact tracers to help my community and make people aware.” (New York City; African American and Black)

Finally, discussions revealed that Latinx/Hispanic participants had faith in institutional authority and trust in local government:

“We have to believe our governor and mayor [of New York].” (New York City; Latinx/Hispanic)

For further categorization of beliefs about contact tracing by the themes identified, please refer to Table 5.

**Table 5:** Beliefs about contact tracing

Theme identified	Example quote from open-ended discussions about contact tracing
Concern with communication method and messenger	“I don’t answer phone calls of people I don’t know. I’d just think it’s a telemarketer or a bill collector.” (New York City, African American)* *“Many times, one stays quiet…or they’re afraid to answer a phone call. They don’t want to give away their privacy. They wouldn’t want to share their information for whatever reason. It could be like a telemarketer or spam.. I think the key is that the person identifies themselves clearly.” (New York, Latinx/Hispanic)* *“People aren’t actually answering no phone. These people think, “Bill collectors are calling right now. I’m not answering.” (New York, African American)* *“If someone said to me, “Would you refer me to a contact tracer, or would you refer me to a doctor or the CDC?” I would refer them to the doctor or the CDC. I would not refer them to someone that’s just hired to get their information and then turn it over to someone else.” (Philadelphia, African American)*
Concerns about surveillance, coercion and privacy	*“It has to do with, like, GPS tracking, like with your phone and your computer, or something like that, something that’s a little bit more technological to it to see literally where you’ve been and who you were in contact with.” (Philadelphia; African American, Latinx/Hispanic and Other Ethnicity)* *“I want to know, is my information going to be private? Are they going to be spread out? I need more information because you really can’t trust anybody…because people sell information and people steal emails. There are a lot of hackers. I would need more information and have more security.”(New York; Latinx/Hispanic)* *“I am skeptical. You’re saying you’re explicitly not sharing it with these two government agencies, it just makes me think of who does get this information and who actually needs it? Privacy is a huge concern for me.” (Philadelphia, African American)*
Mistrust of the government	*“I’m very distrustful at this time of the government and what they’re going to do with information and data.” (Philadelphia; African American, Latinx/Hispanic)* *“I don’t know anybody who would trust that information won’t be shared with immigration agencies because I don’t know that anybody trusts the government much at this point.” (New York City and Philadelphia; African American, Latinx/Hispanic, and White Caucasian)* *“A lot of people are not going to trust [contact tracers] mainly because – although the statement says that the federal government is not going to record your response, a lot of people don’t trust the federal government.” (Philadelphia, African American and Black)*
Fear of punishment and stigma	*“It causes fear…it’s the same thing as if somebody called you in the police precinct saying, ‘Come here, we want to talk to you.’ It’s like the same thing…” (New York City; Latinx/Hispanic)* *“Because people, even though anybody can catch it just like with HIV or whatever, they still would discriminate against you. They would kind of like shun you like you’re a leper or whatever, and they could catch it too.” (Philadelphia, African American, Latinx/Hispanic)* *“You feel afraid to express to somebody, to tell them that you have COVID-19 because you’d think that if you tell him that, they’re going to monitor you, they’re going to lock you in, you won’t be able to go out in the street, you won’t be able to leave your house.” (New York, Latinx/Hispanic)*

^*^Note: Refers to the location and racial and ethnic composition of the focus group

#### Ratings of Concepts

Across groups, participants rated “Be the One” as the top concept with 45% of total first-place votes in the online poll, followed by 30% for “Spread Love,” and 25% for “Keep in Contact.”

**Fig. 1:**
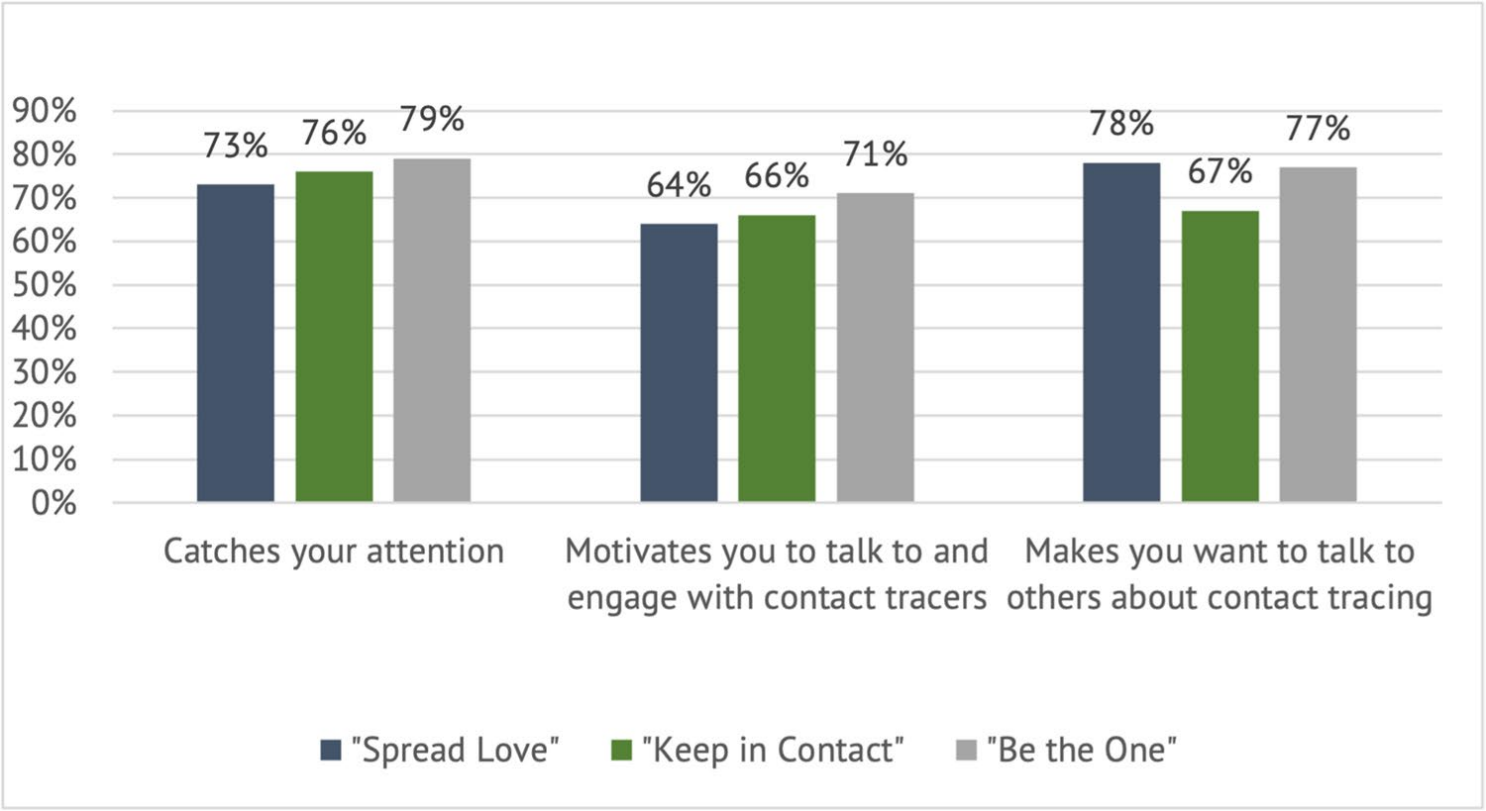
Individual concept reactions

In individual polls (see Fig. 1), “Be the One” received the most positive reactions. 79% of participants reported that “Be the One” caught their attention, followed by “Keep in Contact” (76%) and “Spread Love” (73%). 71% of participants reported the concept “Be the One” motivated them to talk to contact tracers, compared with 66% and 64% of participants for concepts “Keep in Contact” and “Spread Love,” respectively. 78% of participants reported they would talk to others about contact tracing after seeing the concept “Spread Love,” followed by “Be the One” (77%) and “Keep in Contact” (67%).

**Fig. 2:**
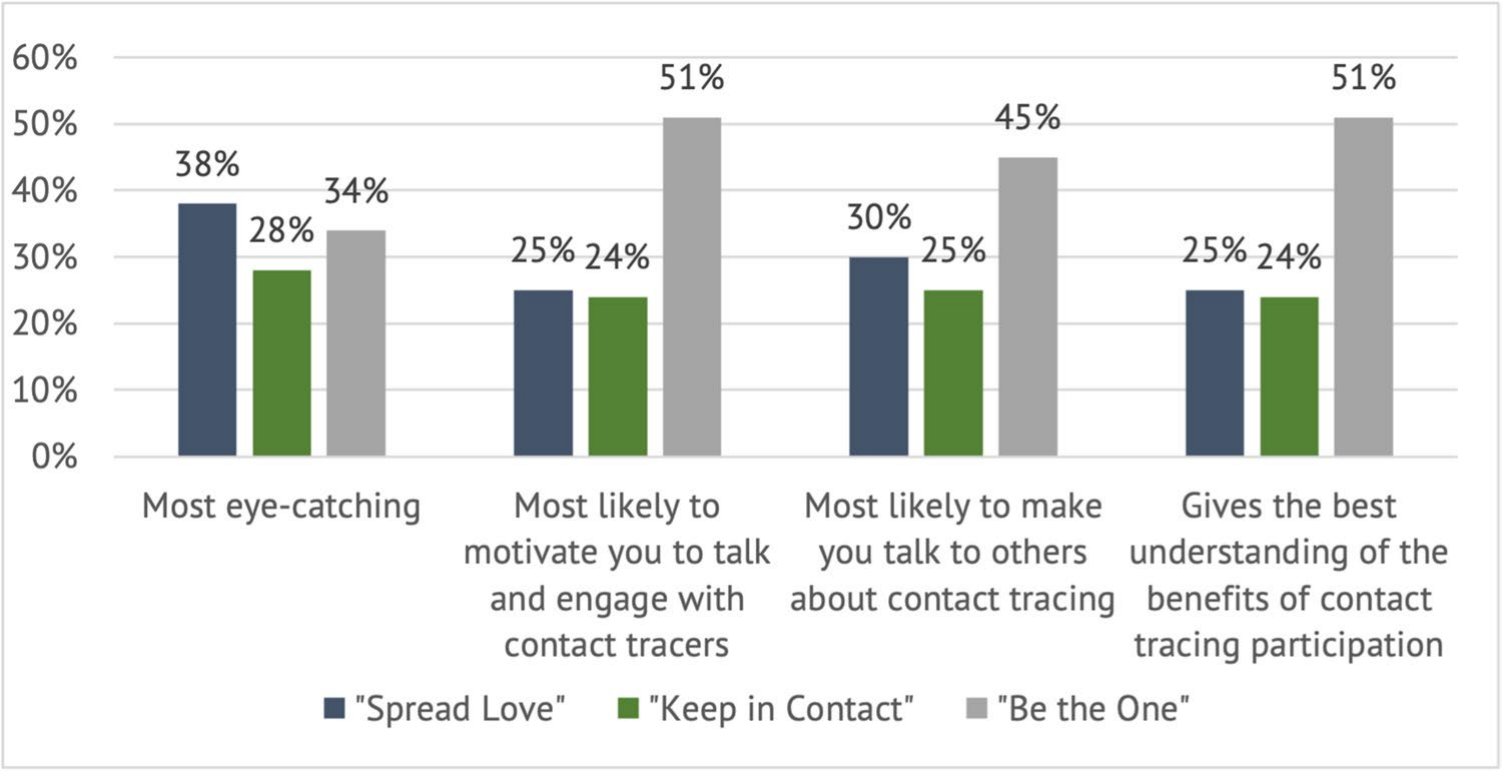
Comparative ratings of concepts on parameters of appeal

In the comparative polls (see Fig. 2), while participants rated “Spread Love” as the most eye-catching (38%), they rated “Be the One” as the best concept for the other parameters of appeal, including most likely to motivate them to talk to and engage with contact tracers (51%) and make them talk to others about contact tracing (45%), and gives the best understanding of the benefits of participating in contact tracing (51%).

### Discussions About the Concepts

Table 3 presents examples of quotes for the concepts and themes discussed below.

#### “Keep in Contact”

##### Attention

Overall, participants said the concept was not very memorable or attention-grabbing. The concept’s emphasis on human and family connection may have eclipsed the message about contact tracing: some participants missed the entire message about contact tracing. While the concept encouraged engagement with contact tracing and discussion with others, many felt that participation in contact tracing was the secondary, and not primary, message.

##### Comprehension

For this concept, participants were able to draw the connection between the benefit of keeping in contact with loved ones and the action of speaking to a contact tracer. However, across groups, participants noted how there was not enough information within the concept to promote or explain what contact tracing was or to answer their questions about COVID-19.

##### Motivation

Participants agreed that the concept sparks conversation but not about contact tracing. The concept’s emphasis on community and family motivated participants to have the intention to work with contact tracers and other public health officials so that they can keep their families and friends healthy and safe from COVID-19.

##### Personal Relevance

Overall, participants found the concept relatable with the imagery reflective of their own experiences dealing with COVID-19 quarantine. The positive community and family-centered imagery, with people that look like their neighbors and families, resonated with participants.

##### Cultural Appropriateness

The concept was consistent with the values shared by participants of the importance of community and staying in touch with family and friends.

#### “Spread Love”

##### Attention

Participants found the concept to be catchy with an easy-to-remember tagline. Participants also had positive reactions to the concept’s images and color presentation.

##### Comprehension

Overall, participants felt that the concept did not provide enough information about COVID-19 and contact tracing. In the current pandemic climate, where most people are taking actions to avoid spreading the disease, for many participants the word “spread” seemed confusing and counterintuitive. Some participants had positive reactions to the concept’s tagline, recognizing the play on words and noting how “spreading” could be turned into a positive. However, many participants said the concept did not successfully connect the benefit of contact tracing and the desired outcome of stopping the spread of COVID-19.

##### Motivation

The concept motivated some participants to consider whether they knew anyone personally who could be a good contact tracer. However, many participants felt the concept did not provide a strong incentive to communicate with contact tracers. Many participants felt that the concept needed a stronger call to action and more specific information in order to motivate the viewer into action.

##### Personal Relevance

Participants connected with the concept and found its imagery relatable. In particular, the concept gave a local feeling because it depicted people that look like people in their community. In addition, participants connected with the concept because the messaging was rooted in the familiarity of their urban communities, such as Philadelphia is known as the city of brotherly love.

##### Cultural Appropriateness

Participants noted that the concept was culturally diverse and shows different demographics. However, participants were divided on how the concept communicated protection and confidentiality of personal information, which is a value shared by participants. Some found it comforting to know that their personal information would be protected. In particular, the concept’s emphasis on personal information remaining confidential made participants feel more comfortable being called by a contact tracer. On the other hand, the concept did not succeed in overcoming the mistrust that some participants had about communicating with an official agency or government-related official.

#### “Be the One”

##### Attention

The direct, active, and engaging tone of the concept was well received by the majority of participants. Participants felt the concept had a sense of urgency that was missing from the other concepts.

##### Comprehension

Participants generally found that the concept provided more information about contact tracing than other concepts. However, when the concept was first introduced, some participants were confused about the concept’s priority audience as they thought it might be a possible recruitment message to hire people to be contact tracers.

##### Motivation

Participants noted that the encouraging and positive messaging of this concept motivated them to speak with contact tracers. They felt that the concept spoke to the self-reliance that they have embraced during the pandemic, encouraging them to take ownership and action and be part of the solution. For other participants, the positive wording and hopeful imagery motivated them to talk with others about contact tracing and eased the perception that participating in contact tracing would be “snitching” on their community or opening themselves up to social ostracization.

##### Personal Relevance

Several participants experienced a personal connection to this concept, perceiving it as communicating the consequences of COVID-19 for their own lives. These participants felt it best captures the seriousness of the pandemic, the immediate need to take action to help stop its spread, and the appropriate tone with which to discuss the issues. In addition, many participants said the concept made them feel like a hero and gave them a sense of duty. Participants expressed that “being the one” can help people feel as if they are doing something useful for their community during this time when so many people feel helpless.

##### Cultural Appropriateness

Despite participants being generally distrustful of the government and having concerns about contact tracing, they said this concept helped to motivate them to participate in contact tracing because it shows they can “be the one” to help reduce the spread of COVID-19 and save lives. The concept seemed to help mitigate participants’ concerns by framing them as the hero and part of the solution, linking the action of participation in contact tracing to the benefit of doing what is needed to prevent the spread of COVID-19 and save lives in their community. However, some participants said the concept could be potentially offensive because it specifically and exclusively targeted certain racial and ethnic groups.

### General Reactions to The Concepts

Overall, participants saw exposure to the idea of contact tracing through the ad concepts as a step in the right direction, starting a conversation that had not been happening previously and encouraging participation that might benefit them and their communities:

“I feel a lot of people aren't familiar with the term [contact tracing] and don't realize how important it really is for them and their communities.” (New York City; African American).

However, while the concepts increased awareness of contact tracing, there was a feeling that some concepts lacked basic and crucial information about COVID-19 and failed to address key practicalities about how contact tracing will work:

“It might be helpful to explain what a contact tracer is for those who may not know.” (Philadelphia; African American, Latinx/Hispanic)

In addition, imagery across concepts, while relatable and inclusive of those who live in their communities, was seen as “too targeted” because they only showed Black and Brown faces, which to some respondents wrongly suggested that people in communities of color were more responsible for the spread of the virus:

“[The campaign] seemed to target a specific demographic as far as race and economic status as if they're the only ones infecting others.”(Philadelphia; African American, Latinx/Hispanic, and Other Ethnicity).

On the other hand, creative executions for each concept that featured local cues (such as local sports teams or personalities) increased relevance with participants, who felt the hometown references customized the concepts to speak directly to them:

“I like how it seems to focus on the brotherly love/sisterly affection theme for Philadelphia.” (Philadelphia; African American)

Overall, after seeing the concepts, many participants saw the benefits of contact tracing and were open to participation:

“Yes [this campaign motivated me to talk to and engage with contact tracers…it helps the community we are from to be cared for and protected.” (New York City; Latinx/Hispanic)

## Discussion

Findings from this study can help public health stakeholders identify key messages and approaches to build trust and engagement in contact tracing efforts for people in communities of color and with low incomes. These insights can be applied to contact tracing efforts during the COVID-19 pandemic, as well as future outbreaks and for other infectious diseases. In addition, although these findings were gathered in the context of communication about contact tracing efforts and were conducted prior to the availability of vaccines for the population in the U.S., insights from this study may be applied to encourage people in communities of color to engage in other public health efforts to prevent the spread of COVID-19, such as vaccinations. While accessibility has been identified as potentially the most significant barrier to vaccines for Black and Latinx/Hispanic people [68, 68], knowledge and attitudes have also been identified as substantial roadblocks [68, 70]. A future study could repeat these focus groups, employing a similar method wherein people’s opinions and concerns are centered to improve communication to support vaccination acceptance and uptake.

Our findings indicate that contact tracing campaigns should amplify personal agency and responsibility to family, friends and community, as demonstrated in previous studies [71, 72]. The highest-rated concept, “Be the One,” shifted message testing participants from associating contact tracing with surveillance, coercion and privacy infringement to associating it with personal agency, empowerment and protection of friends, family and community members. With this in mind, contact tracing media campaigns need to include messages and imagery of personal agency and responsibility to family, friends and community.

This study also showed the importance of ensuring that contact tracers are local, building on recent literature on how a trusted community-based workforce is critical during the COVID-19 response [73–76]. Participants said they would be more likely to trust calls from local area codes and from local government officials, including their local health department, than from the federal government. Trusted local organizations can also augment contact tracing efforts. Participants are more likely to answer a contact tracing call from someone they don’t recognize if the effort has been promoted, endorsed, and reinforced by local organizations with demonstrated care and concern for their community—including churches, mosques, neighborhood groups, outreach programs, health centers and schools. In addition, public health departments should explore using localized messaging and visual cues for contract tracing campaigns to increase local relevance.

Findings from our study also revealed that contact tracing needs to be better explained. Few participants have firsthand experience of contact tracing or an understanding of what it is, how it works, and who it will help. Public health agencies must do a better job explaining contact tracing, carefully crafting messages to debunk myths and model success stories. In the U.S., public opinion polls found low levels of awareness of contact tracing among Black and Latinx/Hispanic adults compared with White adults, and those who had seen or heard little or nothing about it were least likely to say they would be comfortable with the contact tracing process for COVID-19 or likely to engage with it [77]. People need to be reassured that any identifying information will be kept secure and will not be shared with their neighbors, or with housing authorities, immigration authorities, law enforcement, or insurance providers. Several participants said the most suitable contact tracers would be health workers of color and people “who understand them and look like them,” because they can be empathetic and engender a much higher level of trust. With that said, contact tracing campaign messages and images need to be diverse as well as inclusive. Inclusion of non-Black and Brown people in imagery could help alleviate fears expressed by participants of racially motivated targeting.

Finally, our study demonstrated the importance of cultural competence, empathy, and reassuring people about confidentiality. With recent evidence pointing to low levels of trust in public institutions and privacy concerns about technology-based solutions to contact tracing, especially among people in communities of color, our study contributes to evidence on how to build trust in contact tracing efforts [78, 79].

## Conclusion

This study finds that contact tracing efforts should amplify agency and self-determination, better explain contact tracing, and use local contact tracers reinforced by trusted community organizations. Cultural competence, empathy and assurance about confidentiality are also critical.

## Data Availability

Data can be made available upon request and subject to a data use agreement.

## Footnotes

1 People who identified as African or Afro-Caribbean descent.

2 Refers to the location and racial and ethnic composition of the focus group.
